# Our Bureau of Information

**Published:** 1912-01-06

**Authors:** 


### OUR BUREAU OF INFORMATION.

### Our List of Approved Homes.

We have had many applications for insertion in our list of approved homes, and with regard to some of them the inquiries are not yet complete. It would have been convenient to have postponed publishing names for a week or two, for the holiday season has naturally delayed things; but we undertook that the first list should be published in this week's issue. We have therefore only one name to give :—

" Clovelly," Kedleston Road, Derby. Home for undeveloped children and mild mental cases. Principal, Miss Martin, fully certificated nurse. The home is at the extreme verge of the town, and overlooks parks and fields. The children's nurseries are quite apart from the rooms occupied by adult patients.

### Brotherhood Work.

### SOME OF OUR CASES.

We find our Bureau much appreciated by those who are at a loss to know how to help persons in whom they are interested. We also find that many people are willing to help, but do not know how. The would-be helpers are of two kinds—those who can give time and little acts of thoughtfulness and care, and those who can give only money. We would divide them into two classes—honorary helpers, who give of their means, and ordinary ones, who give of themselves. And for almost every case that comes before us there is need of the help of both. In so far as we can we answer queries from the office, but it is impossible for us to know details of every corner of the country, and we call upon those who wish to help their fellow-men to come forward and say what they can do.

An account of one or two of the cases which have been brought before us may serve to indicate the lines of help. Case A is that of a woman worker who finds herself suddenly disabled through hæmorrhage of the lungs. She had worked hard and was earning a good salary, but she had, as duty bade her, helped other members of her family, and had borne the burden of educating younger brothers and sisters. But in the midst of her career her health has broken down. Those she has worked for are as yet only able to maintain themselves, and though they are willing to help to the utmost of their power they cannot give enough to provide the rest and treatment required. For she should first go into a nursing home, by preference in some mild climate, and then have a long period of rest. It is estimated that £30 would be required to make up a sufficient income for her. We are asked what we can do to help. All we can do is to put the case before our readers, and ask if they will join us in aiding this worker to regain even a moderate measure of health. Money is needed, but we want more than money. There is a possibility of a free home being found—at least for a time; but help is needed for maintenance. And wherever she may go there is friendly kindness needed—an occasional visit, a gift of flowers or of some home-made dainty which is not supplied institutionally. Are there members of the Brotherhood to help?

Again there is case B. Case B is a boy with a discharging ear of long standing, due to disease of the middle ear. His mother is a deserted wife, and no one knows where his father is. The mother has gone into domestic service, but can spare no more from her wages than 3s. 6d. a week. This she is now paying to a foster-mother, who is doing her best for the child, and this she is willing to continue paying under any circumstances. She has no wish to shirk her duty. But the doctor who has been treating the boy says that he must have " good food, good air, and daily treatment under medical supervision." And these things cannot be had, especially in the East End of London, for 3s. 6d. a week. We know of a healthy country home under a trained nurse where he could be placed at a very moderate rate, but she cannot take him so cheaply as that, for she too has a living to earn. Can anyone help this boy? The mother is doing all she can. The doctor thinks that with a few years of good feeding and treatment the disease may be cured. This means the difference between health and competence on the one hand, and incurable deafness on the other to the child. Who will help?

Money is not the only essential. In one case which appealed to us we were able, through a correspondent, to tell our inquirer where skilled nursing could be found for a helpless old man. The family did not need pecuniary assistance, but they could not afford to pay a private nurse's fee, and knew not where to obtain the help that was needed. Nothing was needed but—an important but—a member of the Brotherhood in the neighbourhood of the case. He advised, and the need is met.

One helper offers free lodging in her house in a pleasant watering-place to someone who needs rest and change. This she can give, but no more. But it is a great deal. Are there not others who could spare a little house-room for the poor and ailing? If so, let them offer it. We shall send no unsuitable person, no case which is not truly necessitous, and yet, so far as we can ascertain, socially acceptable. We have appeals from all classes. Can we find helpers ready to receive all? Let them offer what they have, be it much or little.

### How to Make the Bureau a Success.

Everyone who uses or wishes to help our Bureau of Information must be kind enough to observe the following rules:

(1) Postal replies cannot be given. Exception will only be made in very special cases at the Editor's discretion.

(2) Queries or answers relating to different cases must be written, or preferably typed, on separate sheets of paper, and the writing must never be crossed.

(3) Questions and replies received not later than Wednesday will, as far as possible, be dealt with in our issue of the following Saturday week.

(4) Letters must have attached the name and address of the sender, with a pseudonym for publication if preferred.

(5) All applicants for insertion in our list of Approved Homes must send a registration fee of five shillings. The omission of this leads to delay in the insertion of names.

(6) *All communications* for this department *must be accompanied by the coupon* to be found at the bottom of the third page of the cover on the inside, and should be addressed to the Editor of the Bureau of Information, c/o The Hospital, 29 Southampton Street, Strand, London, W.C.

Notice.—We invite workers of special experience and knowledge to offer help in replying to questions relating to their special department.

### Needs and Helpers.

"E. L. C." writes: Can you help me to find a home for a boy, five and a-half years, who is suffering from disease of the middle ear of about three years' standing, accompanied by discharge. The mother is a deserted wife, and is in domestic service. She has been paying 3s. 6d. a week to a respectable woman to take care of the child, and the latter has done her best, but cannot provide what is needed—good food, good air, and daily treatment under medical supervision. If these could be provided for a year or two the case might, the doctor thinks, be cured. Is there any home—by preference under a trained nurse—where the boy could be taken at this rate of payment?

"Marie" writes: I offer the use of a large bed-sitting room in my house at Felixstowe to any poor and deserving person who wants rest and a change for a short time. My servant keeps the room clean and does cooking, but occupants must board themselves and find their own light and firing. I am a trained nurse. I also take paying guests and convalescents at £2 2s. a week.

